# Fabrication and Characterization of Polysaccharide Composite Films from Polyion Complex Particles

**DOI:** 10.3390/polym12020435

**Published:** 2020-02-13

**Authors:** Makoto Yamazaki, Kazutoshi Iijima

**Affiliations:** 1 Department of Chemistry, Chemical Engineering and Life Science, College of Engineering Science, Yokohama National University, Tokiwadai 79-5, Hodogaya-ku, Yokohama 240-8501, Japan; yamazaki-makoto-sp@ynu.jp; 2 Faculty of Engineering, Yokohama National University, Tokiwadai 79-5, Hodogaya-ku, Yokohama 240-8501, Japan

**Keywords:** polysaccharide, thin film, polyion complex, glycosaminoglycan

## Abstract

Biomaterials made of natural polysaccharides have attracted much attention due to the fact of their excellent properties, such as high biocompatibility and biodegradability, and their specific biological functions based on their chemical structures. This study demonstrates that polysaccharide composite films can be fabricated from polyion complexes (PICs) with their particles used as building components. Dispersion of PIC particles prepared by mixing, centrifugation, and re-dispersion of dilute solutions of cationic and anionic polysaccharides were cast, dried, and formed into films several micrometers thick. These films were homogenous and water insoluble. It was revealed that the component anionic polysaccharides affected the film’s properties such as the swelling behavior and mechanical characteristics. Adhesion of NIH3T3 cells (integrin: high, CD44: lack or weak) and A549 cells (integrin: high, CD44: high) to the composite films were examined. Both NIH3T3 and A549 cells adhered to heparin/chitosan (HEP/CHI) film because HEP has an affinity for integrin through fibronectin. However, A549 cells adhered to chondroitin sulfate (CS)/CHI and hyaluronic acid (HYA)/CHI films, whereas NIH3T3 cells did not, because both CS and HYA have affinity for CD44. These results indicated that the biological functions of anionic polysaccharides were maintained on the surface of the composite films. It was also possible to fabricate films composed of three kinds of polysaccharides: one cationic polysaccharide and two kinds of anionic polysaccharides. These results show that the properties of films composed of three kinds of polysaccharides may be controllable depending on the anionic polysaccharide composition rates.

## 1. Introduction

Biomaterials made of natural polysaccharides have attracted much attention due to the fact of their excellent features, such as high biocompatibility and biodegradability, and their specific biological functions based on their chemical structures [1,2]. Chitosan (CHI) is a cationic polysaccharide derived from chitin by deacetylation and is easily dissolved in acidic solutions such as acetic acid and hydrochloric acid. In addition to its solubility, it has biodegradability and reactivity [3,4]. Glycosaminoglycans (GAGs), such as heparin (HEP), chondroitin sulfate (CS), and hyaluronic acid (HYA), are each elements of the extracellular matrix (ECM) and interact with cell membrane receptors. For example, CS and HYA have affinity for CD44 which is a cellular receptor [5,6], and HEP interacts with integrin through fibronectin [7]. In order to obtain water-insoluble materials from water-soluble polysaccharides, formation of polyion complexes (PICs) has been widely used. Water-insoluble PICs formed from cationic and anionic polysaccharides by electrostatic interactions can be obtained without modification to other functional groups having polymerizable or hydrophobic regions [8,9,10] and by using cross-coupling agents [11,12]. Polyion complexes are able to adopt various shapes such as thin films, vessels, and bulk solids [13,14,15]. Uniform, smooth films cannot be obtained by casting gel-like PICs [16]. Therefore, other techniques, such as layer-by-layer (LbL) assembly [17,18] and a hot-press technique [19,20], have been developed for fabrication of uniform and smooth films made of polysaccharide PICs. In the LbL assembly technique, substrates are immersed alternately in cationic and anionic aqueous solutions of polysaccharides and result in the formation of polysaccharide composite films [17,18]. It requires protracted periods of time to fabricate films which are several tenths of a micron thick. In the hot-press technique, gel-like PICs are hot-pressed and formed into films [19,20]. By this method, films can be obtained within 30 min and are applicable as drug carriers [21] and cell scaffolds [22]. However, it is difficult to prepare films containing several anionic polysaccharides because of the differences in reactivity between cationic and anionic polysaccharides. Therefore, facile treatments for fabricating free-standing films several microns thick and composed of three or more polysaccharides have not been established.

**Figure 1:**
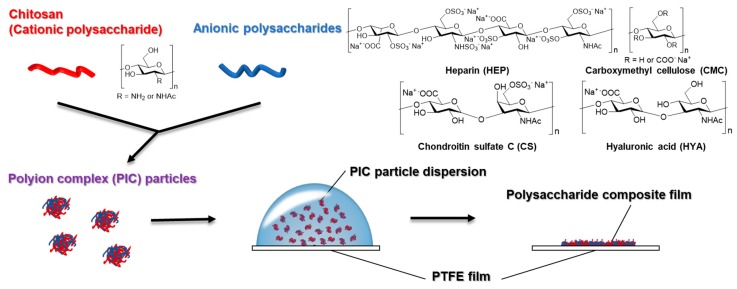
Schematic illustration of the preparation of polysaccharide composite films by casting and drying polyion complex (PIC) particle dispersion.

In this study, we developed a new methodology to fabricate polysaccharide composite films by casting and drying PIC particles (Figure 1). In contrast to gel-like PICs, PIC particles are easily dispersed and can mix uniformly. The PIC particles prepared by mixing aqueous dilute solutions of cationic and anionic polysaccharides were cast, dried, and formed into films on polytetrafluoroethylene (PTFE) sheets. The material properties, swelling behavior, and mechanical characteristics of such films were evaluated. To examine the affinity of anionic polysaccharides for cell membrane receptors, cell adhesion behavior on films was observed. Finally, we attempted to fabricate films composed of three kinds of polysaccharides in order to regulate their properties. Considering the results obtained, the effects of anionic polysaccharide species are discussed.

## 2. Materials and Methods

### 2.1. Materials

Chitosan (CHI), small flakes from crab shell (*M*_W_ ≥ 100,000 Da), heparin sodium salt from pig intestinal mucosa (HEP, *M*_W_ 12,000–20,000 Da), chondroitin sulfate C sodium salt from shark cartilage (CS, *M*_W_ ca. 20,000 Da), and carboxymethyl cellulose sodium salt derived from wood pulp (CMC, *M*_W_ ca. 135,000 Da) were purchased from Nacalai Tesque Inc. (Kyoto, Japan). Hyaluronate sodium salt from *Streptococcus zooepidemicus* (HYA, *M*_W_ 1,200,000–2,200,000 Da) was obtained from Kewpie Corp. (Tokyo, Japan). All reagents were used without further purification. Ultrapure water (18.2 MΩcm) used for the experiments was prepared using Direct-Q UV5 (Merck Millipore, Burlington, MA, USA).

### 2.2. Cell Culture

The NIH3T3 cells (RIKEN Bioresource Center, Tsukuba, Japan) and A549 cells (American Type Culture Collection, Manassas, VA, USA) were maintained in standard Dulbecco’s modified Eagle’s medium (DMEM, Nacalai Tesque) supplemented with 10% (vol./vol.) fetal bovine serum (FBS, Thermo Fisher Scientific, Waltham, MA, USA), 100 U/mL penicillin, and 100 µg/mL streptomycin (Nacalai Tesque) at 37 °C under a humidified 5% CO_2_ atmosphere.

### 2.3. Preparation of PIC Particles and Dispersion

Aqueous solutions of CS, HYA, and CMC (1.0, 2.0, and 1.0 mg/mL as sodium salts, respectively) were added to 1.0 mg/mL CHI solution in 0.1% (vol./vol.) aqueous acetic acid in a dropwise manner with stirring at 700 rpm on a hot-plate stirrer (HPS-2002; AS ONE Corp., Osaka, Japan) and maintained for 15 min at 25 °C. In addition, an aqueous solution of HEP (1.0 mg/mL as sodium salts) was added to 1.75 mg/mL CHI solution in 0.175% (vol./vol.) aqueous acetic acid in a dropwise manner with stirring at 700 rpm for 15 min at 25 °C. After stirring, solutions were centrifuged (4640× *g*, 10 min, Model 5200; KUBOTA Corp., Tokyo, Japan) at 25 °C in order to remove large gel-like PICs. The supernatant was re-centrifuged (21,130× *g*, 30 min, Model 3700; KUBOTA Corp.) at 25 °C and the supernatant was removed. Ultrapure water was added, and the PIC particles were re-dispersed for 15 s using a probe sonicator (VP-050N; Taitec Corp., Saitama, Japan). 

### 2.4. Measurement of PIC Particle Diameters and ζ-potentials 

Diameters and *ζ*-potentials of the PIC particles were measured in phosphate buffered saline (PBS, pH 6.9) using a *ζ*-potential and particle size analyzer (ELSZ-2; Otsuka Electronics Co., Ltd., Osaka, Japan). Each measurement was performed in triplicate, and the results obtained are shown as the mean ± standard deviation (SD).

### 2.5. Preparation of Films Composed of Two Kinds of Polysaccharides

The dispersions were cast on PTFE sheets (Naflon; 100 µm thickness, AS ONE) and dried at 37 °C in an incubator (BNS-110; ESPEC Corp., Osaka, Japan). Hereafter, the PIC particles and films of CS, HEP, HYA, and CMC with CHI are abbreviated as CS/CHI, HEP/CHI, HYA/CHI, and CMC/CHI particles and films, respectively.

### 2.6. Film Characterization 

The morphology of the films was evaluated using scanning electron microscopy (SEM, SU8010; Hitachi High Technologies Corp., Tokyo, Japan) employing an acceleration voltage of 4 kV. The specimens were coated with Pt–Pd using a magnetron sputter (MSP-1S; Vacuum Device Inc., Ibaraki, Japan) to prevent charge-up of the samples. The thickness of the films was calculated from SEM images using ImageJ [23]. Data were obtained for at least ten points and are shown as the mean ± SD. Fourier transform infrared (FT-IR) spectra of CHI powder, a protonated CHI film, each anionic polysaccharide powder, and polysaccharide composite films were obtained by a single reflection attenuation total-reflection (ATR) method using an FT-IR 6200 combined with an ATR PRO450-S (JASCO Corp., Tokyo, Japan). To obtain a protonated CHI film, 10 mg/mL CHI solution in 1% (vol./vol.) aqueous acetic acid were cast on the PTFE films and dried for 24 h at 37 °C.

### 2.7. Film Swelling Behavior

The swelling behavior of the films was evaluated according to the methods described in a previous study [20]. The films were immersed in ultrapure water, incubated at 25 °C, and the degree of swelling was calculated using Equation (1) every 10 min. The equilibrium swelling ratio was defined as the average of swelling ratios when at maximum and ten minutes before and after maximum.

(1) $$\mathrm{Degree}\;\mathrm{of}\;\mathrm{swelling}\;\left(\%\right)=\frac{W_{s}-W_{I}}{W_{I}}\times 100$$

In this equation, *W_I_* and *W_S_* are the initial weights of the film before immersion and the weight of the swelled film after immersion, respectively.

### 2.8. Tensile Strength Measurement

The mechanical properties of the films were evaluated quantitatively using a universal tester (EZ Test EZ-LX; Shimadzu Corp., Kyoto, Japan). First, the film thickness needed to calculate tensile strength (MPa) was calculated from SEM images using ImageJ. The films were then cut into strips and set in the apparatus while maintaining the initial gauge length. The stretching speed was set to 1 mm/min. The obtained stress–strain curves were analyzed using Trapezium X software (Shimadzu Corp.). Young’s modulus of the films was calculated from the slope of the stress–strain curve in strain area 0 [-] to 0.01 [-]. Maximum tensile strength (MPa) and Young’s modulus (MPa) are expressed as means ± SD (*n* = 3).

### 2.9. Adhesion and Proliferation of Cells on Polysaccharide Composite Film

The films were cut using a commercial hole puncher (6.0 mm diameter) and then used in cell cultures. The cut films were sterilized by UV irradiation (254 nm, 4.9 W, 2 h, Toshiba GL-15; Toshiba Ltd., Tokyo, Japan) and then immersed in D-MEM (+) at 37 °C for 1 h in order to swell the films just before cell seeding. The swollen films were set into each well of a 96 well tissue culture-treated PS (TCPS) plate (Sigma–Aldrich, MO, USA). The NIH3T3 and A549 cells dispersed in D-MEM (+) were seeded into each well with the film and the 96 well TCPS plate (200 µL × 1 × 10^4^ cells/per well). The cells were cultured at 37 °C for up to 3 days. After washing with PBS to remove unattached cells, the adherent cells were fixed with 20% (vol./vol.) formalin in PBS containing 10% (vol./vol.) glycerol for 1 h. The cells cultured on polysaccharide composite films and TCPS were stained with Mayer’s hemalum solution (Merck KGaA, Darmstadt, Germany) for 30 min. After washing, cells were observed using an optical microscope (Olympus BX50; Olympus Corp., Tokyo, Japan).

### 2.10. Preparation of Films Composed of Three Kinds of Polysaccharides

To prepare polysaccharide composite films made of three kinds of polysaccharides, two kinds of the dispersed PIC particles were mixed by vortexing, and dispersions were formed into films in the same manner.

## 3. Results

### 3.1. Preparation and Characterization of PIC Particles

After mixing dilute aqueous solutions of cationic and anionic polysaccharides, the Tyndall effect was observed. First, large gel-like PICs were removed by centrifuging the mixed solution. The quantity of large gel-like PICs was <20% of the total amount of polysaccharides (data not shown). Then, PIC particles were collected by centrifugation and re-dispersed with a probe sonicator. The diameters and ζ-potentials of the PIC particles in PBS (pH 6.9) are shown in Table 1. The diameters of PIC particles were several hundred nanometers for all kinds of particles (HEP/CHI, CS/CHI, HYA/CHI, and CMC/CHI). The HEP/CHI, CS/CHI, and CMC/CHI particles showed positive ζ-potentials, and that of HYA/CHI was near-neutral.

### 3.2. Characterization of Films Composed of Two Kinds of Polysaccharides

**Figure 2:**
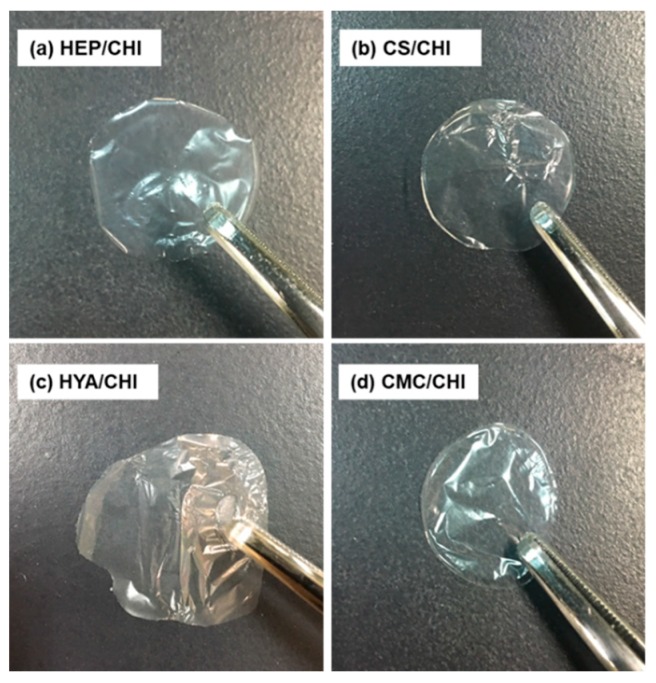
Macroscopic images of the polysaccharide composite films made of HEP/CHI (**a**), CS/CHI (**b**), HYA/CHI (**c**), and CMC/CHI (**d**).

**Figure 3:**
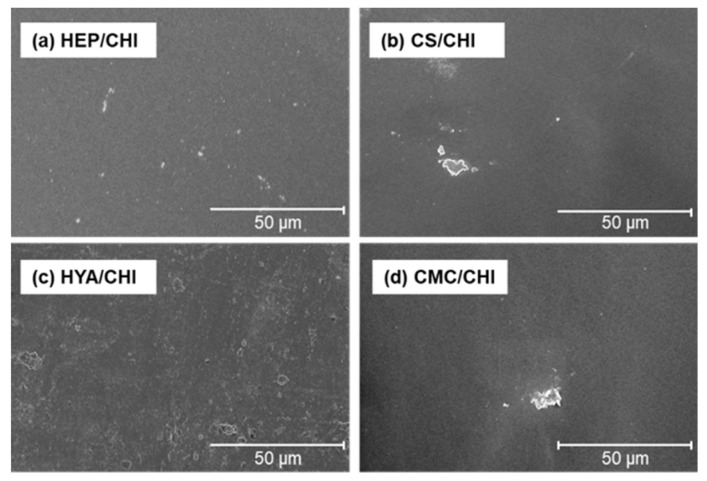
Scanning electron microscopic (SEM) images of polysaccharide composite films composed of HEP/CHI (**a**), CS/CHI (**b**), HYA/CHI (**c**), and CMC/CHI (**d**).

**Figure 4:**
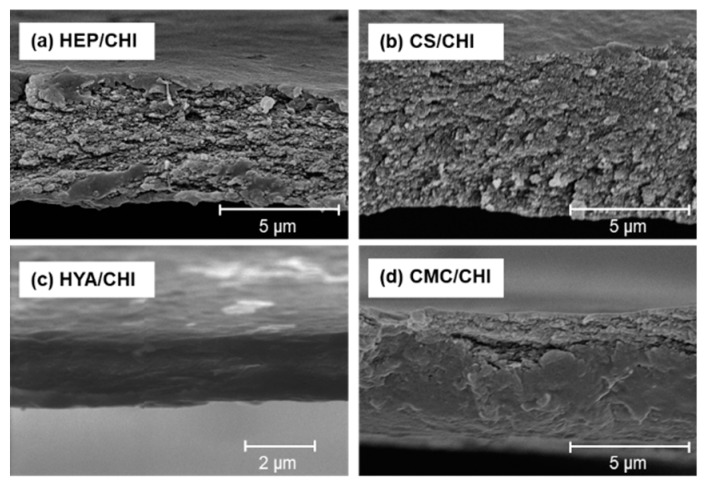
Cross-sectional SEM images of polysaccharide composite films composed of HEP/CHI (**a**), CS/CHI (**b**), HYA/CHI (**c**), and CMC/CHI (**d**).

By casting dispersion of polysaccharide PIC particles and drying at 37 °C, transparent and free-standing films were successfully obtained (Figure 2). All films were macroscopically homogeneous. The SEM observations revealed that the surface structures of polysaccharide composite films were smooth (Figure 3). Cross-sectional images showed that all films had dense structures (Figure 4). Table 2 presents the thickness of the films.

**Figure 5:**
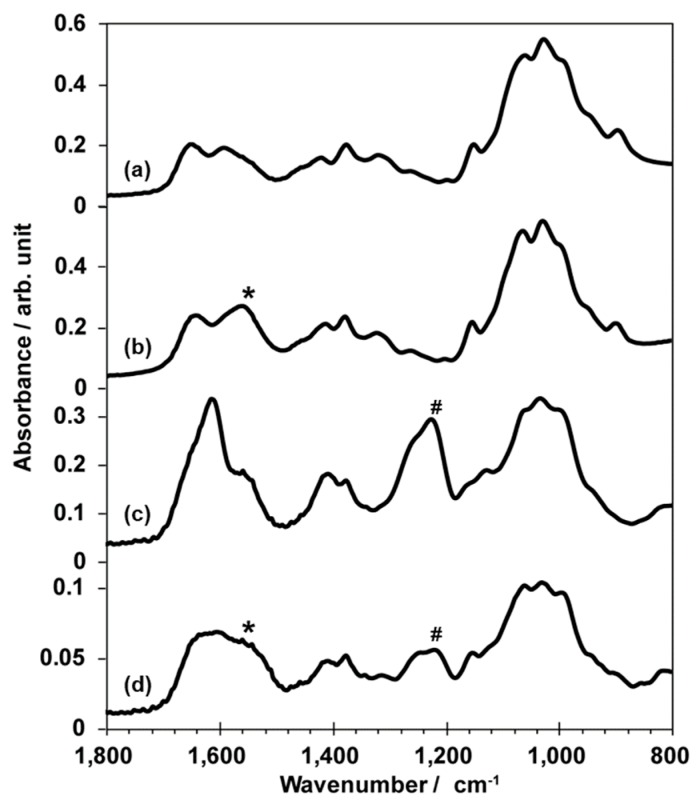
FT-IR spectra of CHI powder (**a**), a protonated CHI film (**b**), CS powder (**c**), and CS/CHI film (**d**). An asterisk (*) and a pound (#) show the position of the peak originating from –NH_3_^+^ and –SO_3_^−^, respectively.

Figure 5 illustrates the FT-IR spectra of CHI powder, a protonated CHI film, CS powder, and CS/CHI film. In addition to peaks derived from carbohydrate frameworks, CHI and CS powder exhibited peaks corresponding to C=O stretching vibration (ca. 1602 cm^−1^, amide I), N–H stretching vibration (ca. 1547 cm^−1^, amide II), and –NH_2_ scissor vibration (ca. 1592 cm^−1^) (Figure 5a,c) [14,24,25]. On the other hand, a protonated CHI film exhibited a peak corresponding to –NH_3_^+^ symmetric vibration (ca. 1558 cm^−1^, * in Figure 5b) [14,24,25] and not –NH_2_ scissor vibration. CS showed specific bands at 1220 cm^−1^ corresponding to –SO_3_^−^ asymmetric stretching vibration (# in Figure 5c) and –COO^−^ symmetric stretching (ca. 1406 cm^−1^) [19,26]. For CS/CHI film, the peak (ca. 1557 cm^−1^) corresponding to –NH_3_^+^ (* in Figure 5d) [14], the peak (ca. 1220 cm^−1^) corresponding to –SO_3_^−^ (# in Figure 5d), and the peak (ca. 1220 cm^−1^) corresponding to –COO^–^ also appeared. Similar peaks originating from –NH_3_^+^, –SO_3_^−^, and –COO^−^ were also observed in HEP/CHI, HYA/CHI, and CMC/CHI films (Figure S1).

All films became soft but did not dissolve after immersion in ultrapure water (data not shown). Table 3 shows the equilibrium swelling ratio (%) of the polysaccharide composite films in ultrapure water. The order of the equilibrium swelling ratio was HYA/CHI > CMC/CHI > HEP/CHI = CS/CHI.

Table 4 shows the tensile strength and Young’s modulus of the polysaccharide composite films. The CS/CHI film had the highest tensile strength of the four kinds of polysaccharide composite films. The order of Young’s modulus was HYA/CHI > CS/CHI = CMC/CHI > HEP/CHI.

### 3.3. Adhesion of Cells to Polysaccharide Composite Films

**Figure 6:**
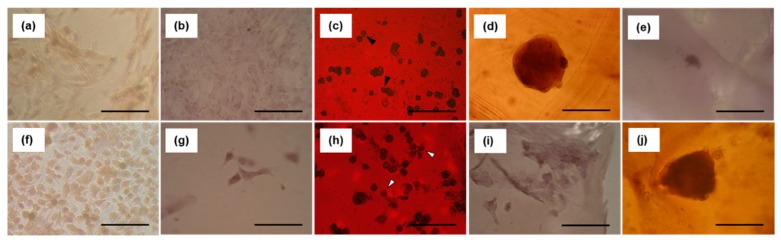
Adhesion of NIH3T3 and A549 cells to polysaccharide composite films and tissue culture-treated polystyrene (TCPS) plates after 3 days of culturing. Microscopic images of NIH3T3 cells (**a**–**e**) and A549 cells (**f**–**j**) seeded on TCPS plate (**a**,**f**), HEP/CHI film (**b**,**g**), CS/CHI film (**c**,**h**), HYA/CHI film (**d**,**j**), and CMC/CHI film (**e**,**k**) after 3 days of culturing. Scale bar: 100 µm. Closed arrowheads in (**c**) and open arrowheads in (**h**) indicate round-shaped and stretched cells, respectively.

The adhesion of NIH3T3 mouse embryonic fibroblast cells and A549 human lung carcinoma cells to polysaccharide composite films was evaluated (Figure 6). Three days after seeding, NIH3T3 cells adhered to the HEP/CHI film and were stretched, with spindle cell morphology (Figure 6b), similar to those on TCPS plates (Figure 6a). The NIH3T3 cells adhered to the CS/CHI film but did not spread (Figure 6c, closed arrowheads). The NIHT3T cells were observed to be aggregated on the HYA/CHI film (Figure 6d). The NIH3T3 cells neither adhered nor spread on the CMC/CHI film (Figure 6e). In contrast, A549 cells on the CMC/CHI film were rounded in shape (Figure 6j), whereas those on the HEP/CHI (Figure 6g), CS/CHI (Figure 6h, open arrowheads), and HYA/CHI (Figure 6i) films were adherent and stretched, with spindle cell morphology, similar to those on the TCPS plates (Figure 6f).

### 3.4. Preparation and Characterization of Films Composed of Three Kinds of Polysaccharides

**Figure 7:**
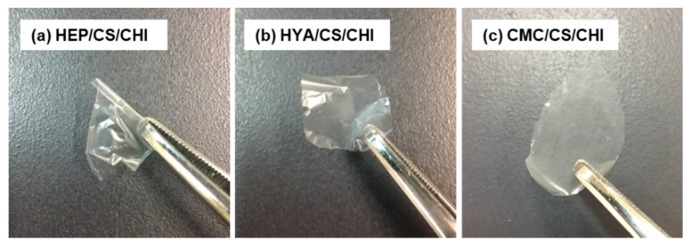
Macroscopic images of polysaccharide composite films made of HEP/CS/CHI (**a**), HYA/CS/CHI (**b**), and CMC/CS/CHI (**c**).

Polysaccharide composite films of HEP/CS/CHI, HYA/CS/CHI, and CMC/CS/CHI were also successfully fabricated by casting of a mixture of two kinds of PIC particle dispersions. All films were transparent and free-standing (Figure 7). The FT-IR spectra identified peaks representing −NH_3_^+^ symmetric vibration (ca. 1520 cm^−1^) [14,24,25] and −SO_3_^–^ asymmetric stretching vibration (ca. 1220 cm^–1^) [19] were observed not only in CS/CHI film (* and # in Figure 5d) but also in HYA/CS/CHI film (* and # in Figure S1f). Furthermore, chemical composition analysis using X-ray photoelectron spectroscopy (XPS) revealed that HYA/CS/CHI film contained S in addition to C, N, and O (Table S1). In HYA/CS/CHI films, the atomic ratio of sulfur (0.78%) was between that of CS/CHI films (1.01%) and HYA/CHI films (0.00%).

## 4. Discussion

The basic concept of this study was to fabricate polysaccharide composite films from PIC particles which are otherwise used as building components (Figure 1). At first, monodispersed PIC particles were prepared by mixing, centrifugation, and redispersion of dilute aqueous solutions of both cationic and anionic polysaccharides. The PIC particles were formed from CHI with HEP [27], CS [28], HYA [29], and CMC [30]. It was revealed that centrifugation and redispersion of PICs of polyelectrolytes allowed for monomodal size distribution [31]. By combining these processes, PIC particles of CHI with HEP, CS, HYA, and CMC were prepared. Since PDI values between 0.1 and 0.3 show a narrow width size distribution [32], it was indicated that the monodispersed PIC particles were successfully obtained (Table 1). The monodispersed PIC particles may contribute to form homogeneous films.

Isolated PIC particles were cast, dried, and transformed into transparent and homogenous films (Figure 2). The PIC particles were formed based on electrostatic interactions between cationic and anionic polysaccharides and were dispersed stably due to the repulsion of surface charges [33]. By drying the solvent, concentrations of dispersed PIC particles increased. Further drying resulted in formation of transparent films (Figure S2; note that a video S1 is also available in the Supplementary Materials). All films became soft but did not dissolve after immersion in ultrapure water (data not shown). Such irreversible behavior indicated the recombination and integration of polymer networks among particles during drying. Surface and cross-sectional SEM images of the films under dry conditions indicated dense and smooth structures (Figure 3 and Figure 4). The surface of CS/CHI film was also analyzed by AFM (Figure S3). The fact that the root mean square (RMS) calculated from AFM images was 25.7 nm supported the smoothness of the film surface. The fabrication of such smooth films by casting large PIC gels is difficult because of their extensive structures. The thickness of the films reported in this study was approximately several micrometers (Table 2). Because the films were composed of PIC particles, the thickness of films might be controllable by increasing the concentration of dispersion.

The chemical composition of the films was characterized using FT-IR. Compared to the FT-IR spectra derived from N–H stretching vibration (ca. 1547 cm^−1^, amide II) and the –NH_2_ scissor vibration (ca. 1592 cm^−1^) of CHI powder, the peak corresponding to –NH_3_^+^ (ca. 1558 cm^−1^) was detected in a protonated CHI film. The CS was characterized by bands at 1220 cm^−1^ and 1406 cm^−1^ due to the –SO_3_^−^ asymmetric stretching vibration and –COO^−^ symmetric stretching [19,26]. In the CS/CHI film, bands appeared at 1558 cm^−1^, 1220 cm^−1^, and 1406 cm^−1^. These absorptions can be attributed to –NH_3_^+^, –SO_3_^−^ and –COO^−^ which supported PICs formation involving –NH_3_^+^ and –SO_3_^−^ or –COO^−^ groups of anionic polysaccharides [14,24,25,26]. From the FT-IR spectra (Figure 5 and Figure S1), it was concluded that the fabricated films were composed of two kinds of polysaccharides. 

The effects of anionic polysaccharides on the characteristics of the composite films were also investigated. The equilibrium swelling ratio of the composite films depended on the kinds of anionic polysaccharides involved. It is known that HYA and CMC have excellent water-holding abilities [34,35]. The results that films containing them also showed high equilibrium swelling ratio (Table 3) indicated that anionic polysaccharide properties were maintained in the composite films. To characterize the mechanical properties of the films, tensile strength tests were conducted. All films were less stretchable (Young’s moduli of the films were from hundreds MPa to several GPa). The maximum tensile strengths at film breakpoints are presented in Table 4. The CS/CHI and HYA/CHI films obtained by this method showed higher tensile strengths than those prepared by the hot-pressing technique for the same composition [20]. The order of tensile strengths was HYA/CHI film > CS/CHI film > CMC/CHI film > HEP/CHI film which was not simply correlated with the molecular weight, charge density, and equilibrium swelling behavior. These results indicated that the molecular weight and chemical structure of anionic polysaccharides combined affected the nature of PICs which resulted in differences in mechanical properties of the films. 

Next, interactions between composite films and cells via membrane receptors were analyzed. It is well known that GAGs have specific affinity for cells through membrane proteins and ECM. Among anionic polysaccharides used in this study, HEP has an affinity for integrin through fibronectin [7], and CS and HYA have an affinity toward CD44 [5,6]. In contrast, CMC lacks affinity for these cellular membrane proteins. To examine whether anionic polysaccharides in our composite films maintained such affinities or not, adhesion of two types of cells, NIH3T3 and A549, to the composite films was examined. For NIH3T3 cells, the level of expression of integrin is high, whereas that of CD44 is either absent or weak [36]. In contrast, A549 highly expresses both integrin and CD44 [37]. Both NIH3T3 and A549 cells adhered to the HEP/CHI film, because HEP has an affinity for integrin (Figure 6b,g). However, A549 cells adhered to CS/CHI and HYA/CHI films, but NIH3T3 cells did not (Figure 6c,d,h,i), because both CS and HYA have affinity for CD44. Neither NIH3T3 nor A549 cells were adherent on CMC/CHI films due to the fact of their lack of affinity for these membrane receptors (Figure 6e,j). These results indicated that the biological functions of anionic polysaccharides were retained on the surfaces of the composite films. In addition, CD44 is also known as a mesenchymal stem cell (MSC) marker [38]. It might be possible to isolate MSCs from cell mixtures, such as bone marrow and adipose tissues, on films composed of CS and HYA which have affinity. Because of their biocompatibility and degradability, films with isolated MSCs can be directly transplanted into patients.

Finally, fabrication of films composed of three kinds of polysaccharides was accomplished. Homogeneous dispersions were successfully prepared without aggregation by mixing two kinds of dispersions because the particles in the dispersions had the same ζ-potentials (Table 1). When oppositely charged dispersions of PIC particles were mixed, PIC particles became aggregated (data not shown). The films were thin and transparent as was the case with films composed of two kinds of polysaccharides. It was also suggested from FT-IR spectra (Figure S1) and XPS analysis (Table S1) that the films were composed of three kinds of polysaccharides. This indicated that our technique could combine CHI and two or more kinds of any anionic polysaccharides in order to fabricate films.

## 5. Conclusions

Free-standing and water-insoluble polysaccharide composite films with homogeneous and smooth surface structures were obtained by casting and drying PIC particles. It was indicated that these films exhibited characteristics based on the kinds of anionic polysaccharides incorporated. The films have sufficient tensile strength for practical use, and have water-insoluble properties which are desirable for biomaterials. Furthermore, it is important to note that our method is applicable to preparing films composed of three kinds of polysaccharides. Because it has been reported that PICs of polysaccharide composites show good material loading and release abilities [21], the potential of polysaccharide composite films as vehicles for growth factors and/or loaded with drugs and as implantable cell scaffolds is suggested. Furthermore, it is also suggested that the fabrication of films from polysaccharide PIC particles, otherwise used as building components, can also be applied for the construction of composite polysaccharide films involving other materials such as biominerals (e.g., hydroxyapatite and tricalcium phosphate), fillers (e.g., cellulose nanofibers), and functional nanomaterials (e.g., gold nanoparticles and magnetic nanoparticles).

## Figures and Tables

**Table 1:** Diameter and ζ-potential of PIC particles in PBS (pH 6.9).

Composition	Diameter/nm	PDI/-	ζ-Potential/mV
HEP/CHI	680.9 ± 23.5	0.264	40.15 ± 1.55
CS/CHI	491.5 ± 43.6	0.199	26.47 ± 0.23
HYA/CHI	791.4 ± 95.0	0.303	0.08 ± 1.05
CMC/CHI	519.4 ± 11.9	0.257	21.97 ± 2.14

**Table 2:** Thickness of polysaccharide composite films.

Film	Thickness/μm
HEP/CHI	4.38 ± 0.44
CS/CHI	5.35 ± 1.53
HYA/CHI	1.76 ± 0.32
CMC/CHI	5.65 ± 1.52

**Table 3:** Equilibrium swelling ratio of polysaccharide composite films.

Film	Equilibrium Swelling Ratio/%
HEP/CHI	149 ± 22.6
CS/CHI	138 ± 30.5
HYA/CHI	725 ± 110.9
CMC/CHI	412 ± 31.5

**Table 4:** Mechanical strength and Young’s modulus of polysaccharide composite films.

Film	Tensile Strength/MPa	Young’s Modulus/MPa
HEP/CHI	11.8 ± 0.4	154.5 ± 76.2
CS/CHI	121.6 ± 56.0	877.9 ± 254.2
HYA/CHI	296.5 ± 69.0	6662.2 ± 1615.3
CMC/CHI	14.1 ± 4.0	687.3 ± 223.6

## Supplementary Materials

The following are available online at https://www.mdpi.com/2073-4360/12/2/435/s1, Figure S1. FT-IR spectra of powder of HEP (a), HYA (d), and CMC (g) and composite films made of HEP/CHI (b), HYA/CHI (e), CMC/CHI (h), HEP/CS/CHI(c), HYA/CS/CHI (f), and CMC/CS/CHI (i). An asterisk (*) and a pound (#) symbol show the position of the peaks originating from -NH_3_^+^ and -SO_3_^−^, respectively. Figure S2. Time-lapse imaging of the formation of the CS/CHI composite films from CS/CHI PICs particle dispersion: 0 min (a), 80 min (b), 160 min (c), 240 min (d), and 320 min (e). A movie is also available as supplementary data. Figure S3. Typical 3D (a) and 2D (b) atomic force microscopic images and surface profiles (c) of CS/CHI film surface from an approximately 2 µm × 2 µm scanned area. Table S1. Atomic fractions of elements detected on the surface of CS/CHI, HYA/CHI, and HYA/CS/CHI films by XPS. Video S1. Time-lapse movie of the formation of the CS/CHI composite films from CS/CHI PICs particle dispersion.
