# Supplementary material for: Fabrication and Characterization of Polysaccharide Composite Films from Polyion Complex Particles
Source: Polymers (Basel). 2020 Feb 13;12(2):435. doi: 10.3390/polym12020435 (PMC7077694; doi:10.3390/polym12020435)
Supplement: Supplementary file 1 [file polymers-12-00435-s001.zip › polymers-718392.SI.Proof done/polymers-718392..SI.pdf]

polymers

Supplementary Materials

Article

Fabrication and Characterization of Polysaccharide Composite Films from Polyion Complex Particles

Makoto Yamazaki [1] and Kazutoshi Iijima [1,2,*]

[1] Department of Chemistry, Chemical Engineering and Life Science, College of Engineering Science, Yokohama National University, Tokiwadai 79-5, Hodogaya-ku, Yokohama 240-8501, Japan; yamazaki-makoto-sp@ynu.jp
[2] Faculty of Engineering, Yokohama National University, Tokiwadai 79-5, Hodogaya-ku, Yokohama 240-8501, Japan
* Correspondence: iijima-kazutoshi-mh@ynu.ac.jp; Tel.: +81-45-339-3997

1. Atomic force microscopic observation of the film

Atomic force microscopy (AFM) was carried out in air on an SPI3800N/SPA-400 platform (Seiko Instruments Inc., Chiba, Japan). An Au-coated 200-mm long soft cantilever with integrated pyramidal silicon nitride tips (spring constant 0.02 Nm^{-1}) (SN-AF01-S-NT, Seiko Instruments Inc.) was used. A topographic image was taken in contact mode, with a scan rate of 2 Hz. Data visualization and analysis were conducted using Gwyddion software [1].

2. X-ray photoelectron spectroscopy

The chemical compositions of CS/CHI, HYA/CHI and HYA/CS/CHI films were analyzed by X-ray photoelectron spectroscopy (XPS, PHI Quantera II, ULVAC-PHI, Inc., Kanagawa, Japan). The energy scale of the instrument was calibrated by measuring the Au 4f7/2, Ag 3d5/2, and Cu 2p3/2 photoelectron lines at 84.0, 368.3, and 932.7 eV, respectively, for pure metal foils. Spectra were recorded while irradiating the sample with a monochromatic Al Ka source (*hn* =1486.6 eV) operating at 24.2 W. The beam diameter was 100 mm. Elements present on the surface were identified from survey spectra recorded over the energy range 0-1100 eV at intervals of 1 eV and a pass energy of 280 eV. High-resolution spectra were recorded for selected photoelectron peaks (C 1s, O 1s, N 1s, and S 2p) at intervals of 0.2 eV and a

pass energy of 224 eV. Obtained data were analyzed using the PHI MultiPak software (ULVAC-PHI, Inc.) in order to determine the elemental composition.

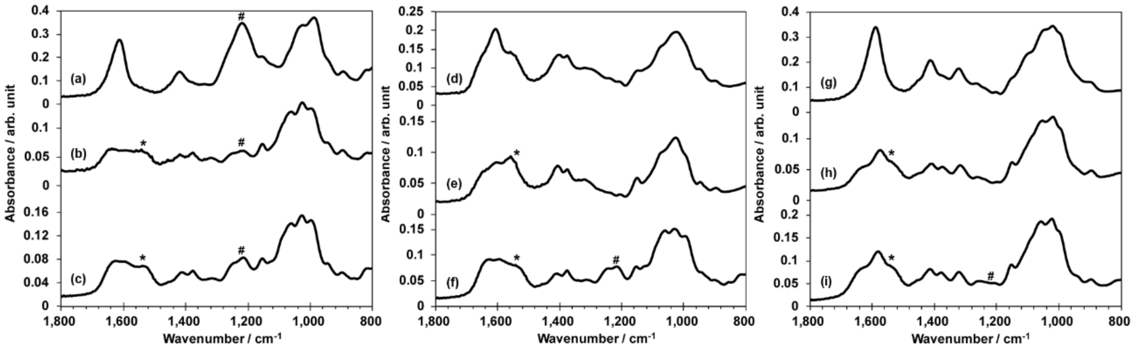

Figure S1. FT-IR spectra of powder of HEP (a), HYA (d) and CMC (g) and composite films made of HEP/CHI (b), HYA/CHI (e), CMC/CHI (h), HEP/CS/CHI(c), HYA/CS/CHI (f) and CMC/CS/CHI (i). An asterisk (*) and a pound (#) show the position of the peak originated from $-NH_3^+$ and $-SO_3^-$, respectively.

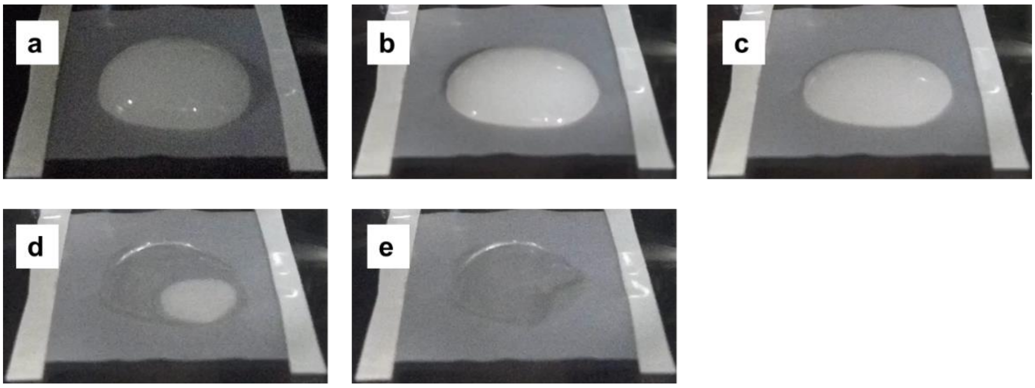

Figure S2. Time-lapse imaging of formation of the CS/CHI composite films from CS/CHI PICs particle dispersion. 0 min (a), 80 min (b), 160 min (c), 240 min (d), and 320 min (e). A movie is also available as a Supplementary data.

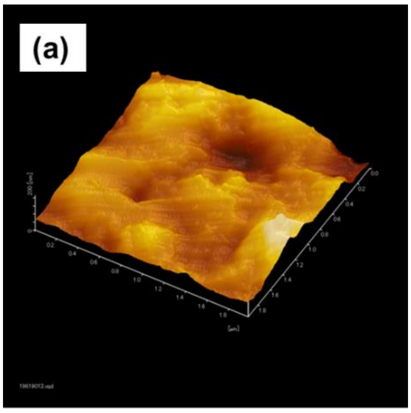

Figure S3. A typical three-dimensional (3D) (a) and 2D (b) atomic force microscopic (AFM) images and surface profiles (c) of CS/CHI film surface from approximately 2 μm × 2 μm scanned area.

Table S1. Atomic fractions of elements detected on the surface of CS/CHI, HYA/CHI and HYA/CS/CHI films by XPS.

Film	C / %	O /%	N / %	S / %
CS/CHI	60.74	32.99	5.26	1.01
HYA/CHI	72.68	26.57	0.76	0.00
HYA/CS/CHI	67.57	26.60	5.05	0.78

Reference

[1] Nečas, D.; Klapetek, P. Gwyddion: an open-source software for SPM data analysis. *Cent. Eur. J. Phys.* **2012**, *10*, 181–188. [10.2478/s11534-011-0096-2]
